# Effects of one-time alternate-row deep fertilization on yield, quality, and nitrogen use efficiency in wheat following rice

**DOI:** 10.3389/fpls.2025.1687010

**Published:** 2025-12-16

**Authors:** Yuting Zhang, Canping Dun, Guanghui Shi, Shijie Yan, Lei Yan, Can Zhao, Hongcheng Zhang, Weiling Wang, Zhongyang Huo

**Affiliations:** 1 Jiangsu Key Laboratory of Crop Genetics and Physiology, Yangzhou University, Yangzhou, China; 2 Jiangsu Key Laboratory of Crop Cultivation and Physiology, Yangzhou University, Yangzhou, China; 3 Jiangsu Co-Innovation Center for Modern Production Technology of Grain Crops, Yangzhou University, Yangzhou, China; 4 Agricultural College, Yangzhou University, Yangzhou, China

**Keywords:** yield, slow-release nitrogen fertilizer, deep application methods, nitrogen use efficiency, protein content

## Abstract

**Introduction:**

Deep placement technology has gradually become a key direction for simplified cultivation. However, few studies have explored the effects of different deep-band placement under the application of slow-release nitrogen fertilizer (SRNF) combined with urea on wheat yield and quality.

**Methods:**

In this study, four treatments were designed: conventional split application of urea (CK), one-time broadcasting of SRNF combined with urea (M1), and two different one-time deep-band placement treatments of SRNF combined with urea (M2 and M3).

**Results:**

The results showed that compared with CK and M1, M2 could increase the nitrogen content in rhizosphere soil after the jointing stage, which in turn affected the activities of nitrogen assimilation enzymes and promoted nitrogen uptake and utilization in the aboveground parts of wheat. In addition, the M2 maintained a relatively high leaf area index and net photosynthetic rate, ultimately increasing the post-anthesis dry matter accumulation and laying a material foundation for yield improvement. The wheat yield under the M2 was significantly by 4.8% higher than that of the CK, which was mainly attributed to the increase in spike number and grains per spike. In contrast, M3 could maintain a stable yield while reducing grain protein content, thereby improving the quality of weak-gluten wheat.

**Discussion:**

This study provides a theoretical basis and practical guidance for the development of deep fertilization technology for wheat following rice.

## 1 Introduction

The middle and lower reaches of the Yangtze River are one of the major growing areas for wheat following rice in China (Jin et al., 2023; Ladha et al., 2003; Tong et al., 2014). In this region, the average nitrogen (N) application rate during the wheat season reaches 300–400 kg ha^-1^, which is 1.5–2 times the actual N requirement of wheat, while the apparent N use efficiency is only 30%-35% (Grant et al., 2012; Li et al., 2012). Traditional split broadcast application of N fertilizer not only involves high labor intensity and challenges in timing control, but also fails to deliver N precisely to the root zone (Hong et al., 2021; Song et al., 2023; Wan et al., 2021). This limitation restricts wheat’s absorption and utilization of N and conflicts with the requirements of modern simplified cultivation (Zheng et al., 2023; Zhu et al., 2021). Therefore, optimizing N management strategies for rice-wheat systems in this region is critically important.

To address this issue, one-time fertilization techniques have emerged. Slow-release N fertilizer (SRNF), due to its long release period and the characteristic that nutrient release is synchronized with the crop’s demand, makes it possible to realize one-time fertilization while ensuring the crop yield (Dun et al., 2024). Among them, the combined application of SRNF and urea has been proven to improve the yield and N use efficiency of wheat following rice in the middle and lower reaches of the Yangtze River, as it can balance nutrient supply in the early stage and sustained N release in the middle and late stages (Xiao et al., 2024; Zhang et al., 2024b). Xiao et al. (2024) found that compared with the split application of urea, the one-time broadcasting of a mixture of SRNF and urea at a ratio of 7:3 significantly increased the yield and N fertilizer utilization rate of wheat after rice. In addition to the ratio of SRNF to urea, existing studies have confirmed that compared with N fertilizer broadcasting, under the condition of deep N fertilizer application, the yield of crops such as wheat and rice can be increased by 7.57%-11.85%, and the apparent N recovery efficiency is 46.37%-58.57% higher than that of broadcasting (Chen et al., 2021; Liu et al., 2023). Although the advantages of deep N fertilizer application are widely recognized, there are relatively few studies on the regulatory effects of combined deep application of slow-release urea and urea on the growth of wheat following rice.

The commonly used deep fertilization method for wheat is deep-band placement, which involves applying N fertilizer in a concentrated band in the soil below and to the side of the seeds (Qiang et al., 2022; Wang et al., 2023a; Wu et al., 2021). Previous studies have carried out extensive research on the effects of different fertilization depths, as well as the deep-band placement of slow-release urea combined with urea, on wheat yield, N use efficiency, and other related indicators (Akhtar et al., 2025; Wang et al., 2022). Typically, there is one fertilizer row on each side of every wheat row (one row of fertilizer serving one row of plant in total, RR1), which can provide nutrients evenly for each row of wheat (Chen et al., 2016; Wang et al., 2022). Although deep fertilization improves fertilizer use efficiency compared with broadcasting, it increases the operational intensity of machinery, imposes higher requirements on the quality of equipment, and results in higher fuel consumption and increased costs (Kargbo et al., 2016; Zhu et al., 2019). If the number of fertilizer rows can be reduced, the operational intensity of machinery can be decreased and the cost input lowered (Song et al., 2023). For example, one fertilizer row can supply nutrients for two rows of wheat (one row of fertilizer serving two rows of plant in total, RR2). However, RR2 can lead to uneven nutrient supply on both sides of wheat rows, posing potential risks of yield reduction or specific quality variation. Currently, there are few studies on the effects of RR2 on wheat yield and quality.

Therefore, a two-year field experiment was conducted to explore the effects of different deep-band placement (RR1and RR2) on the yield and quality formation of wheat after rice stubble and the absorption of N fertilizer. The results would be significant in establishing lower-cost deep-band methods for wheat production in the middle and lower reaches of the Yangtze River.

## 2 Materials and methods

### 2.1 Experimental site and materials

**Figure 1:**
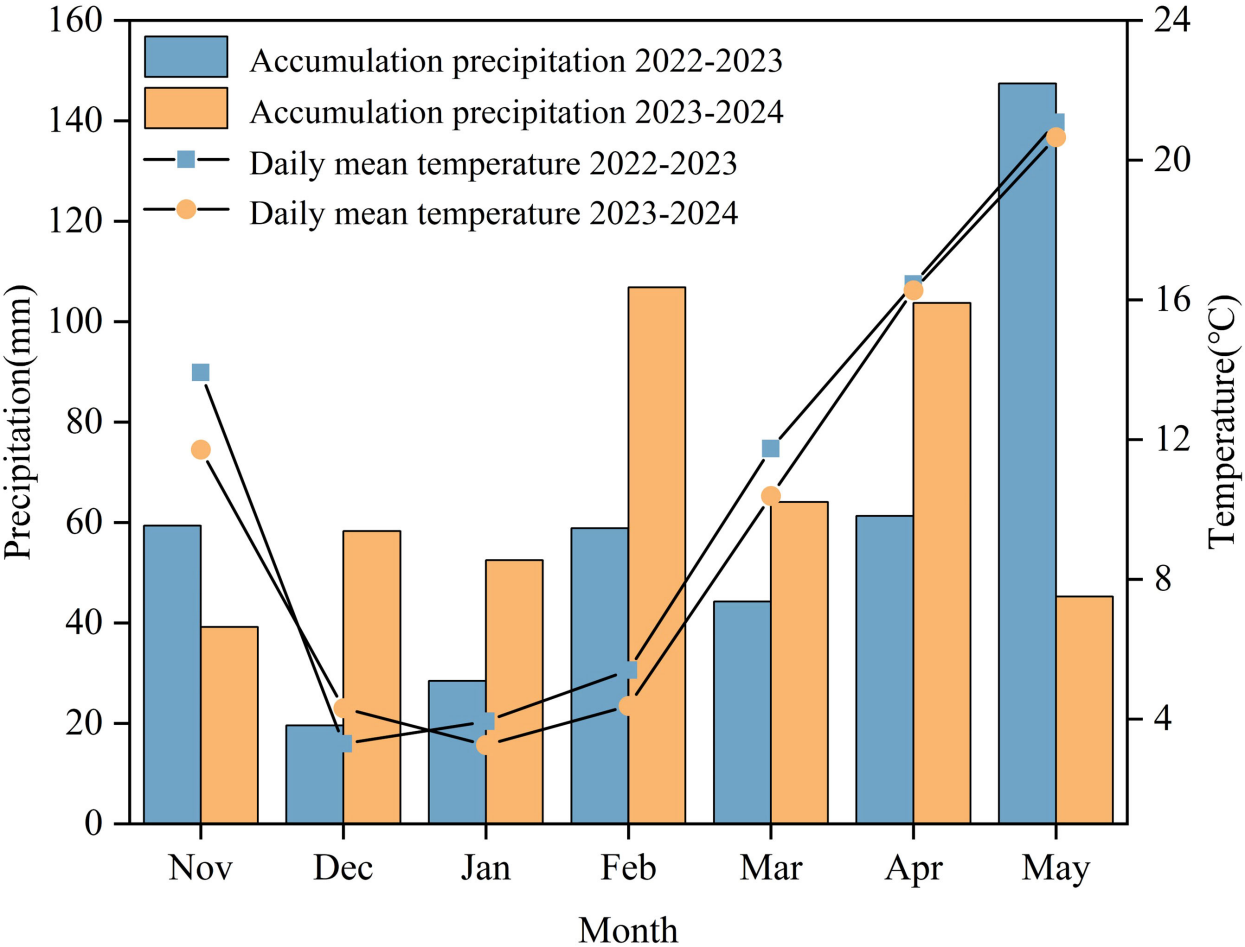
Temperature and precipitation during two wheat growing seasons in 2022-2024.

This experiment was conducted during two wheat growing seasons (2022-2024) at Jingxian Farm, Jiangyan District, Taizhou City (120°10′E, 32°22′N). The experiment site is located in the middle and lower reaches of the Yangtze River. In this region, a crop rotation system has long been practiced, with rice planted in summer (June-October) and wheat planted in winter (November-May of the following year) in rotation. The region has a subtropical monsoon climate (Zhou et al., 2023), and the soil at the experimental site is paddy soil. Before sowing, the main soil properties of the surface soil (0–20 cm) were determined (2-year average), with the results as follows: organic matter content 15.43 g kg^-1^, available N 89.55 mg kg^-1^, available phosphorus 22.75 mg kg^-1^, available potassium 104.51 mg kg^-1^, and pH 6.84. The wheat cultivars used in this experiment were Yangmai 22 (YM22), a weak-gluten wheat cultivar, and Yangmai 39 (YM39), a medium-strong gluten wheat cultivar—both of which are major cultivars in the rice-wheat rotation regions of the middle and lower Yangtze River Basin. The SRNF had a 90-day N release longevity (44% N content; manufactured by Hanfeng Slow-Release Fertilizer Co., Ltd., Jiangsu, China). Common urea (46% N), superphosphate (15% P_2_O_5_) and potassium chloride (60% K_2_O) were purchased from a local fertilizer distributor. The average temperature and cumulative precipitation were 10.83°C and 419.29 mm, respectively, during the 2022–2023 wheat growing season, and 10.14°C and 469.88 mm respectively during the 2023–2024 wheat growing season (**Figure 1**).

### 2.2 Experimental design

**Figure 2:**
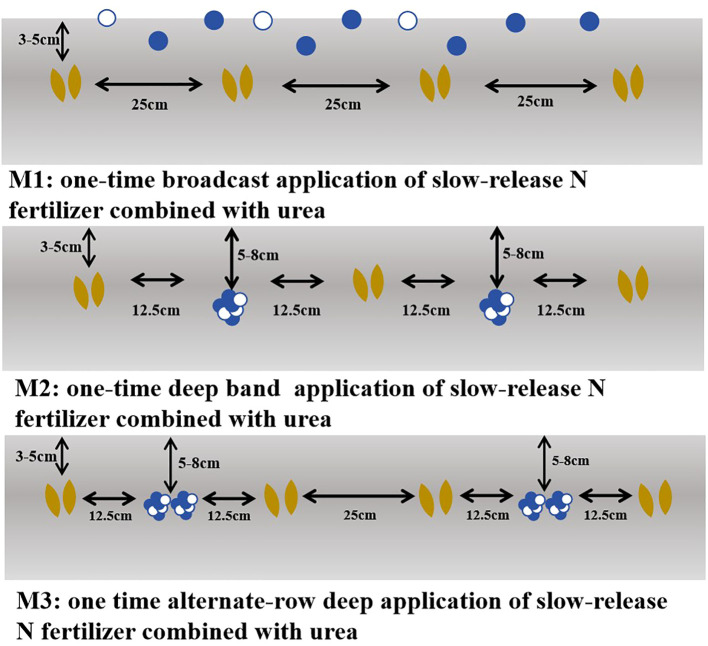
Schematic diagrams of different deep application methods.

This experiment adopted a split-plot randomized block design, with variety as the main plot and fertilization method as the subplot. The split application of urea was used as the control (CK: 50% of the basal fertilizer was broadcast before sowing and then incorporated into the soil by rotary tillage; additionally, 10% as tillering fertilizer, 20% as jointing fertilizer, and 20% as booting fertilizer were all broadcast directly at their respective stages). Additionally, four treatments with different application methods of SRNF combined with urea were established (Based on our previous research (Xiao et al., 2024), a 7:3 ratio of SRNF to urea is the optimal proportion for wheat following rice production. Therefore, we selected this ratio for one-time application). They are as follows: (1) M1: one-time broadcast application of SRNF combined with urea. The fertilizer was evenly spread on the soil surface before sowing; (2) M2: one-time deep band application of SRNF combined with urea. Fertilization was conducted at a depth of 5–8 cm in the soil between wheat plants (at a row spacing of 12.5 cm), with a distance of 12.5 cm from the wheat plants on both sides of each fertilization row; (3) M3: one-time alternate-row deep application of SRNF combined with urea. Fertilization was carried out at a depth of 5–8 cm in the soil in alternate rows (at a row spacing of 25 cm), with a distance of 12.5 cm from the wheat plants on both sides of the fertilization rows, and the row spacing of wheat plants in the unfertilized rows was 25 cm. The detailed design is shown in **Figure 2**. Each treatment was replicated three times, with a plot area of 12 m^2^. The N fertilizer application rate was 240 kg ha^-1^ for all treatments, while the application rates of both phosphate and potassium fertilizers were 135 kg ha^-1^. Meanwhile, a treatment with no N fertilizer application was also set up. Wheat was sown in rows by manual furrowing, with a row spacing of 25 cm and a basic seedling density of 2.25 × 10^6^ plants ha^-1^ across all treatments. No artificial irrigation was conducted during the experiment, and the field management measures after sowing were consistent with local conventional agricultural production practices.

### 2.3 Sampling and measurement

#### 2.3.1 Yield and its component factors

The number of effective panicles per unit area and grains per panicle were investigated before wheat harvest. One 1 m² representative quadrat was selected per plot. The aboveground biomass was collected, air-dried naturally, and then threshed. Grain yield and 1000-grain weight were adjusted to a standard moisture content of 13%.

#### 2.3.2 Stem and tiller number, earing percentage of stem and tillers

At the jointing stage, three 1 m row segments with uniform growth were selected to determine tiller number in the field. Tiller number at the jointing stage represents the maximum stem and tiller number during wheat growth.

Earing percentage of stem and tillers (EPST) (%)=Number of panicles/Maximum stem and tiller number.

#### 2.3.3 Dry matter and N accumulation

At the anthesis and maturity stages, 20 representative plants were sampled from each treatment, and their organs were separated. Samples were placed in an oven at 105°C for 1 h to deactivate physiological activities, then dried at 80°C to constant weight for dry matter accumulation determination. The calculation of parameters related to dry matter accumulation and translocation was performed with reference to the method described by Wang et al. (2025).

Pre-anthesis dry matter remobilization amount (PDRA)=Dry matter accumulation of vegetative organs at anthesis - Dry matter accumulation of vegetative organs at maturity.

Post-anthesis dry matter accumulation (PDMA)=Dry matter accumulation of at maturity (DMM)- Dry matter accumulation of at anthesis (DMA).

Contribution rate of dry matter remobilized pre-anthesis to the grain (CDP) (%)=PDRA/Grain dry weight×100.

Contribution rate of dry matter accumulated post-anthesis to the grain (CDA) (%)=1-CDP.

The Kjeldahl method was used to determine the N concentration of each organ at the anthesis stage and maturity stage. The plant N accumulation was calculated as the sum of the dry matter accumulation of each organ multiplied by its corresponding N concentration. N accumulation and remobilization were calculated f according to the method of Zhang et al. (2022).

Pre-anthesis N translocation (PNT) (kg ha^-1^)=N accumulation in vegetative organs at anthesis stage-N accumulation in vegetative organs at maturity stage.

Contribution rate of Pre-anthesis N translocation to the grain (CPNT) (%)=PNT/Grain N accumulation×100.

Post-anthesis N Accumulation (PNA) (kg ha^-1^)=N accumulation at maturity stage-N accumulation at anthesis stage.

Contribution rate of PNA to the grain (CPNA) (%)=PNA/Grain N accumulation×100.

The N agronomic efficiency (NAE) was calculated according to Ke et al. (2023).

NAE (kg kg^-1^)=(Y_N_-Y_0_)/F_N_.

Y_N_ and Y_0_ are the total yield (kg ha^−1^) at maturity with and without N fertilizer, respectively. F_N_ are the N fertilizer input amount (kg ha^−1^).

#### 2.3.4 Photosynthetic rate and leaf area index

Five flag leaves with uniform growth and synchronized anthesis were selected on sunny days during the anthesis and milk-ripe stages. Between 9:00 and 11:00, the LI-6400 portable photosynthesis system (LI-COR, USA) was used with a red-blue light source in the leaf chamber. Light intensity was controlled at 1200 μmol m^-2^ s^-1^, and leaf chamber CO_2_ concentration was maintained at 400 μmol mol^-1^. The *P_n_* of the leaves was measured, with 3 replicates for each measurement.

At the flowering stage and milk-ripe stage, 20 plants were sampled from each plot. The leaf area was measured using a desktop leaf area meter (LI-3100C, USA). The LAI was calculated by the formula: LAI=(Sample leaf area (cm²)/Number of sampled plants) × Basic seedling density (plants·m^-^²).

#### 2.3.5 Nitrate reductase and glutamine synthetase activity

At the anthesis and milk-ripe stages, wheat flag leaves with healthy and consistent growth were selected, rapidly frozen in liquid N, and then stored at -80°C in an ultra-low temperature refrigerator for the determination of Rubisco activity, nitrate reductase (NR) activity, and glutamine synthetase (GS) activity.

The activities of NR and GS were determined according to Yao et al. (2024). Fresh leaf tissue (0.3 g) was crushed in 0.1 M phosphate buffer (pH 7.5) prepared by mixing NaH_2_PO_4_·2H_2_O and Na_2_HPO_4_·12H_2_O, and the homogenate was centrifuged at 12000×g for 20 min at 4°C. The reaction mixture consisted of NADH, 1.2 mL of 0.1 M KNO_3_, and 0.4 mL of the extraction solution. The mixtures were incubated at 25°C for 30 min. For the control, 0.4 mL of 0.1 M sodium phosphate buffer (pH 7.5) was used instead of NADH. The reaction was terminated by the addition of 1 mL of sulphanilamide, followed by 1 mL of 1% N-1-naphthylethylenediamine dihydrochloride. The red chromophore was allowed to develop for 15 min prior to centrifugation at 12000 × g for 10 min. NR activity was determined using a nitrite N standard curve, with absorbance measured at 540 nm using the supernatant.

GS activity was determined as follows. A fresh sample (0.2 g) was ground in a pre-chilled mortar and pestle, then dissolved in 3 mL of 5 mM sodium phosphate buffer (pH 7.2) containing 50 mM Na_2_SO_4_ and 0.5 mM Na_2_EDTA. The homogenate was centrifuged at 20000×g for 20 min at 4°C. The reaction mixture consisted of 1.2 mL of the extraction supernatant, 0.3 mL of 0.3 M Na-Glu, 0.6 mL of 0.25 M imidazole-HCl (pH 7.0), 0.2 mL of 0.5 M MgSO_4_, and 0.4 mL of 0.03 M Na-ATP (pH 7.0). After incubating at 25°C for 5 min, 0.2 mL of 1.0 M hydroxylamine was added. The reaction was terminated by adding 0.8 mL of a mixed reagent (10% FeCl_3_·6H_2_O, 50% (v/v) HCl, and 24% (w/v) trichloroacetic acid) following a 20 min incubation at 25°C. To measure the absorbance at 540 nm, the mixtures were centrifuged at 15000 × g for 10 min after allowing the red chromophore to develop for 20 min. GS activity was calculated using a γ-glutamyl-hydroxamate standard curve.

#### 2.3.6 Rhizosphere soil inorganic N content

During the overwintering, jointing, booting, anthesis, and maturity stages of wheat, 20 representative plants were selected from each experimental plot. Rhizosphere soil samples were collected using the root-shaking method. After removing loose soil, stones, and root residues, the soil adhering to the root surface was collected with a brush. The soil samples from each plot were thoroughly mixed to prepare a composite sample, which was then placed in a container and stored in a -40°C freezer. After extractiossn with 2 mol/L potassium chloride solution, the concentrations of ammonium N (NH_4_^+^-N) and nitrate N (NO_3_^–^N) in the soil were determined using a continuous flow injection analyzer (Model: AA3-A001-02E, Manufacturer: Bran-Luebbe, Place of Origin: Norderstedt, Germany). Inorganic N content was defined as the sum of NH_4_^+^-N and NO_3_^–^N.

#### 2.3.7 Quality index

The wheat was harvested after it matured, the husks were removed from the wheat ears after threshing, and then the wheat seeds including the seed coat and endosperm were ground into flour to determine the quality indicators. The total N content of flour was determined by the Kjeldahl method (Kong et al., 2024), and the protein content (PC) was calculated using a conversion coefficient of 5.7. The wet gluten content (WGC) was measured with a gluten instrument (Perten Instruments AB, Stockholm, Sweden) according to Zhang et al. (2024a). The grain sedimentation value (SV) was determined by the SDS constant method according to Zhao et al. (2012).

### 2.4 Statistical analysis

A linear mixed model (LMM) analyzed the effects of year, variety, treatment, and their interactions on wheat indicators. Variety, treatment, and their interaction were fixed factors (to quantify deterministic effects on indicators), while year (capturing inter-annual environmental variation) and within-year replicates (accounting for field block errors) were random factors. Type III ANOVA tested fixed effect significance, and multiple comparisons used least squares means (LSMEANS) with the Tukey method. All analyses were conducted in R software (packages: lme4, lmerTest, emmeans). For mean comparison between treatments, Duncan’s multiple range test was used, with statistical significance set at *p* < 0.05 (n=3). Figures were plotted using Origin 2022 (OriginLab Corp., Northampton, MA, USA). Multilinear regression was used to analyze the contribution of yield components to grain yield by SPSS.

## 3 Results

### 3.1 Grain yield

As shown in **Table 1**, variety had a significant effect on spike number and grains per spike; treatment had an extremely significant effect on spike number, grains per spike, and yield, and a significant effect on 1000-grain weight. Compared with CK, M1 significantly reduced wheat yield, with a yield decrease of 5.42% and 5.39% in the 2022–2023 and 2023–2024 growing seasons, respectively. One-time deep application of SRNF combined with urea (M2 and M3) significantly increased wheat yield compared with M1, with yield increases of 12.54% and 4.55% in 2022–2023 and 13.29% and 4.36% in 2023-2024, respectively. Among these treatments, the yield of M2 was significantly higher than those of CK, with yield increases of 6.42% and 7.18% in the 2022–2023 and 2023-2024. However, there was no significant difference in yield between M3 and CK.

**Table 1:** Effects of different deep application methods on wheat yield and its component factors.

Year	Variety	Treatment	Spike number (×10^4^ ha^-1^)	Grains per spike	1000-Grain weight (g)	Yield (×10^3^ kg ha^-1^)
2022-2023	YM22	CK	471.00 ± 4.58ab	36.49 ± 0.74ab	47.62 ± 0.87a	7.65 ± 0.05b
		M1	435.67 ± 9.29c	35.95 ± 0.58b	48.04 ± 0.20a	7.33 ± 0.12c
		M2	480.00 ± 12.49a	37.69 ± 0.92a	47.40 ± 1.04a	8.13 ± 0.08a
		M3	457.00 ± 5.57b	36.55 ± 0.80ab	46.94 ± 0.59a	7.63 ± 0.17b
	YM39	CK	459.33 ± 16.77ab	37.49 ± 1.02ab	46.85 ± 0.85a	7.56 ± 0.14b
		M1	416.00 ± 19.70c	36.22 ± 0.38b	47.52 ± 0.44a	7.06 ± 0.1c
		M2	475.33 ± 7.23a	38.24 ± 0.82a	47.47 ± 2.00a	8.06 ± 0.08a
		M3	447.33 ± 3.79b	37.36 ± 1.15ab	46.77 ± 1.18a	7.42 ± 0.08b
2023-2024	YM22	CK	487.89 ± 10.38ab	37.14 ± 0.76ab	47.34 ± 1.43a	8.19 ± 0.15b
		M1	457.86 ± 6.36c	36.57 ± 0.69b	48.74 ± 0.72a	7.74 ± 0.18c
		M2	496.17 ± 14.06a	38.19 ± 0.52a	47.19 ± 0.62a	8.78 ± 0.19a
		M3	469.02 ± 13.79bc	37.59 ± 0.87ab	47.04 ± 0.61a	8.03 ± 0.17b
	YM39	CK	470.90 ± 11.89ab	38.06 ± 0.48ab	46.94 ± 0.59a	8.05 ± 0.17b
		M1	441.63 ± 8.42c	37.28 ± 0.63b	47.38 ± 1.03a	7.62 ± 0.19c
		M2	487.98 ± 8.88a	39.31 ± 0.72a	47.26 ± 0.36a	8.62 ± 0.12a
		M3	464.24 ± 13.48b	38.11 ± 0.81ab	46.83 ± 0.77a	8.00 ± 0.17b
LMM		V	*	*	ns	ns
		T	**	**	*	**
		V×T	ns	ns	ns	ns

CK, split application of urea; M1, one-time broadcast application of SRNF combined with urea; M2, one-time deep band application of SRNF combined with urea; M3, one time alternate-row deep application of SRNF combined with urea. In LMM results, * and ** indicate the significance of variety (V), treatment (T), and their interactions at the 0.05 and 0.01 levels, respectively; ns indicates no significant effect. Values are means ± standard error (n=3). Different lowercase letters after the same column value indicate significant difference at the level of 0.05.

In terms of yield components, compared with CK, M1 significantly reduced wheat spike number, with decreases of 8.47% and 6.19% in the 2022–2023 and 2023-2024, respectively. M2 and M3 increased both spike number and grains per spike compared with M1. For spike number, the increases were 12.22%, 6.21% in 2022–2023 and 9.43%, 3.78% in 2023-2024, respectively. For grains per spike, the increases were 5.21%, 2.40% in 2022–2023 and 4.94%, 2.51% in 2023-2024, respectively. Among them, the spike number and grains per spike of M2 were higher than those of CK. There were no significant differences in spike number or grains per spike between M3 and CK. No significant difference in 1000-grain weight was observed among all treatments. Multilinear regression analysis of yield components showed that deep application of SRNF combined with urea improves wheat yield primarily by increasing spike number (*r* = 0.76-0.84, *p* < 0.001) and grains per spike (*r* = 0.57-0.76, *p* < 0.05), rather than by affecting 1000-grain weight (*r* = -0.23-0.09, *p* = 0.112-0.407) (**Table 2**).

**Table 2:** Contributions of yield components to grain yield was analyzed by the multilinear regression.

Yield component	Trait	2022-2023		2023-2024	
		YM22	YM39	YM22	YM39
Spike number	*r*	0.83	0.84	0.76	0.78
	*p*	<0.001	<0.001	<0.001	<0.001
Grains per spike	*r*	0.76	0.66	0.57	0.73
	*p*	<0.001	0.004	0.014	0.001
1000-grain weight	*r*	-0.33	0.067	-0.23	0.089
	*p*	0.112	0.407	0.200	0.376

*r* and *p* indicated correlation, and significance, respectively.

### 3.2 Stem and tiller number, EPST

**Figure 3:**
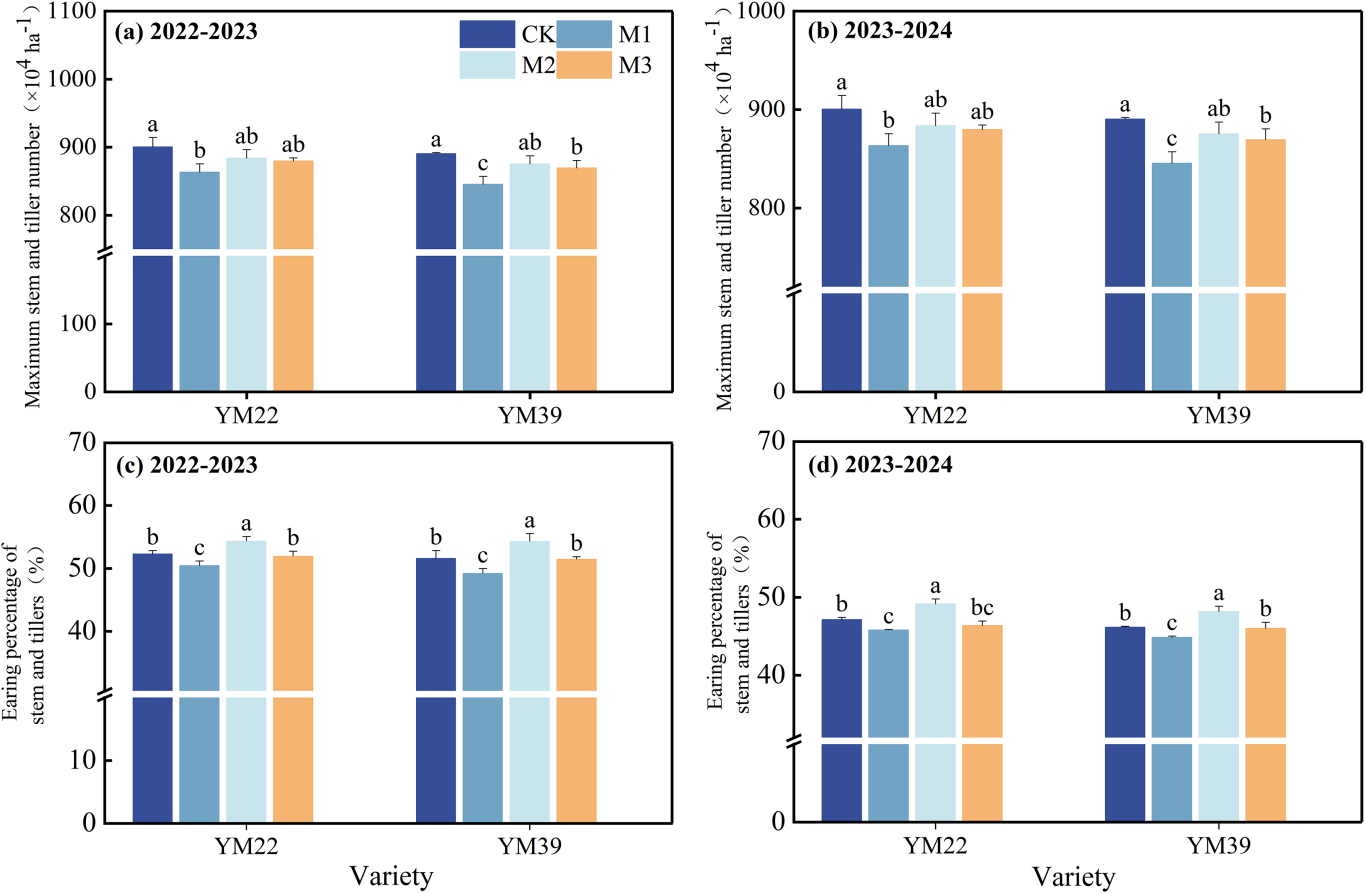
Effects of different deep application methods on maximum stem tiller number and ear production rate of stem tillers. CK, split application of urea; M1, one-time broadcast application of SRNF combined with urea; M2, one-time deep band application of SRNF combined with urea; M3, one time alternate-row deep application of SRNF combined with urea. Vertical bars indicate mean standard errors of three replicates. Different lowercase letters indicate significant difference at 0.05 level.

As shown in **Figure 3**, compared with CK, the maximum stem and tiller number of M1 was significantly reduced, while that of M2 and M3 decreased only slightly. Compared with CK, the EPST of M1 was significantly decreased by 4.10% and 2.81% in the 2022–2023 and 2023-2024, respectively. The EPST of M2 and M3 was significantly higher than that of M1, with increases of 9.03%, 3.78% in the 2022–2023 and 7.36%, 1.92% in the 2023-2024, respectively. There was no significant difference in EPST between M3 and CK, but it was significantly lower than that of M2.

### 3.3 Dry matter accumulation and remobilization

**Figure 4:**
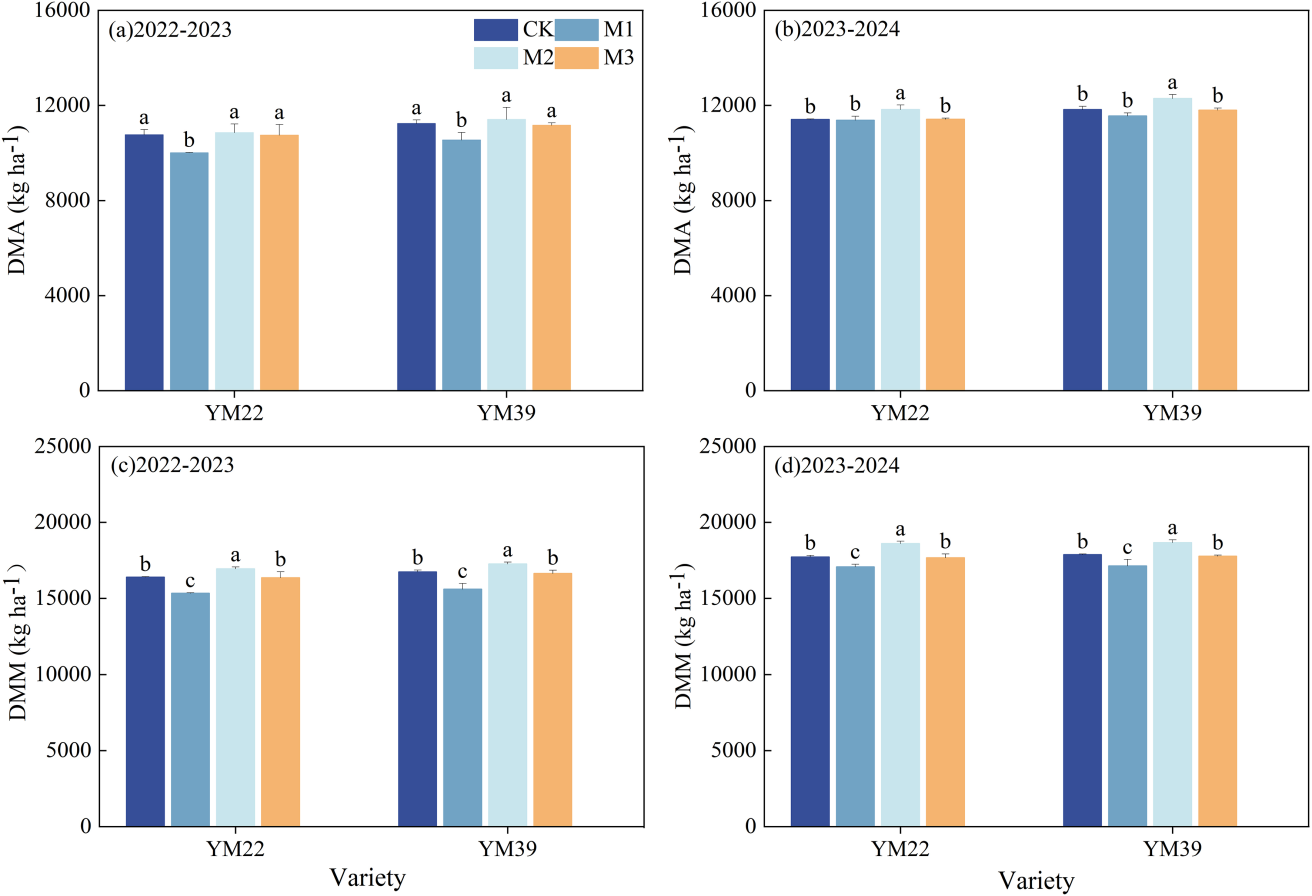
Effects of different deep application methods on dry matter accumulation at anthesis and maturity in wheat. CK, split application of urea; M1, one-time broadcast application of SRNF combined with urea; M2, one-time deep band application of SRNF combined with urea; M3, one time alternate-row deep application of SRNF combined with urea. Vertical bars indicate mean standard errors of three replicates. Different lowercase letters indicate significant difference at 0.05 level.

As shown in **Figure 4**, in the 2022–2023 growing season, the DMA of M1 was significantly lower than that of other treatments, while in the 2023–2024 growing season, the DMA of M2 was significantly higher than that of other treatments. The DMM of M1 was significantly lower than that of CK, with decreases of 1.80% and 3.90% in 2022–2023 and 2023-2024, respectively. Compared with M1, both M2 and M3 significantly increased DMM; the increases in DMM were 5.15%, 1.44% and 8.94%, 3.63% in 2022–2023 and 2023-2024, respectively. Among them, the DMM of M2 was significantly higher than that of CK, and there was no significant difference in DMM between M3 and CK.

As shown in **Table 3**, variety had a significant effect on PDRA and DMPA, while treatment exerted an extremely significant effect on PDRA, CPD, PDMA, and CDA. The PDRA and CPD of M1 were significantly higher than those of other treatments. The PDRA and CPD of M2 were lower than those of CK. Compared with CK, M1 significantly decreased PDMA and CDA, with reductions of 6.71% and 8.75% in PDMA, and 5.00% and 7.64% in CDA in the 2022–2023 and 2023–2024 growing seasons, respectively. Compared with M1, both M2 and M3 significantly increased PDMA, with increases of 14.99%, 6.82% and 16.68%, 8.42% in 2022–2023 and 2023-2024, respectively. The PDMA of M2 was significantly higher than that of CK, with increases of 7.26% and 6.45% in 2022–2023 and 2023-2024, respectively. The PDMA of M3 was significantly lower than that of M2 but showed no significant difference from that of CK. There was no significant difference in CDA between M2 and CK, but the CDA of M2 was significantly higher than that of M3.

**Table 3:** Effects of different deep application methods on wheat dry matter accumulation and remobilization.

Year	Variety	Treatment	Pre-anthesis dry matter remobilization amount (kg ha^-1^)	Contribution rate of dry matter remobilized pre-anthesis to the grain (%)	Post-anthesis dry matter accumulation (kg ha^-1^)	Contribution rate of dry matter accumulated post-anthesis to the grain (%)
2022-2023	YM22	CK	1969.04 ± 55.15b	29.36 ± 0.10bc	5627.95 ± 124.55b	70.64 ± 0.25a
		M1	2103.01 ± 48.77a	33.45 ± 0.69a	5333.71 ± 147.02c	66.55 ± 1.08b
		M2	1995.09 ± 14.95b	28.75 ± 0.10c	6096.72 ± 87.23a	71.25 ± 1.19a
		M3	2020.44 ± 26.23b	32.21 ± 3.37ab	5616.91 ± 170.07b	67.79 ± 0.21b
	YM39	CK	2066.89 ± 2.82b	29.72 ± 0.42b	5509.27 ± 66.37b	70.28 ± 1.10a
		M1	2092.84 ± 31.88a	32.68 ± 0.30a	5057.49 ± 145.59c	67.32 ± 0.11b
		M2	2026.03 ± 7.23b	28.68 ± 0.30c	5850.59 ± 88.45a	71.32 ± 0.10a
		M3	2031.39 ± 28.09b	30.12 ± 0.37b	5478.70 ± 195.37b	68.28 ± 0.60b
2023-2024	YM22	CK	2197.24 ± 29.71b	30.24 ± 1.11bc	6308.31 ± 243.87b	69.76 ± 2.35a
		M1	2493.57 ± 54.30a	35.60 ± 1.21a	5688.72 ± 196.04c	64.40 ± 2.58b
		M2	2171.49 ± 63.17b	28.48 ± 1.16c	6774.28 ± 263.47a	71.52 ± 1.14a
		M3	2256.15 ± 46.70b	32.12 ± 1.91b	6247.18 ± 206.03b	67.88 ± 1.42b
	YM39	CK	2289.36 ± 100.19b	31.25 ± 1.08bc	6042.65 ± 182.26b	68.75 ± 2.21a
		M1	2559.64 ± 56.84a	36.46 ± 1.40a	5579.06 ± 236.98c	63.54 ± 1.80b
		M2	2222.38 ± 112.57b	29.05 ± 1.97c	6375.32 ± 90.55a	70.95 ± 2.69a
		M3	2312.10 ± 90.32b	33.69 ± 2.23ab	5970.90 ± 190.26b	66.31 ± 1.15b
LMM		V	*	ns	*	ns
		T	**	**	**	**
		V×T	ns	ns	ns	ns

CK, split application of urea; M1, one-time broadcast application of SRNF combined with urea; M2, one-time deep band application of SRNF combined with urea; M3, one time alternate-row deep application of SRNF combined with urea. In LMM results, * and ** indicate the significance of variety (V), treatment (T), and their interactions at the 0.05 and 0.01 levels, respectively; ns indicates no significant effect. Values are means ± standard error (n=3). Different lowercase letters after the same column value indicate significant difference at the level of 0.05.

### 3.4 LAI and *P_n_*

**Figure 5:**
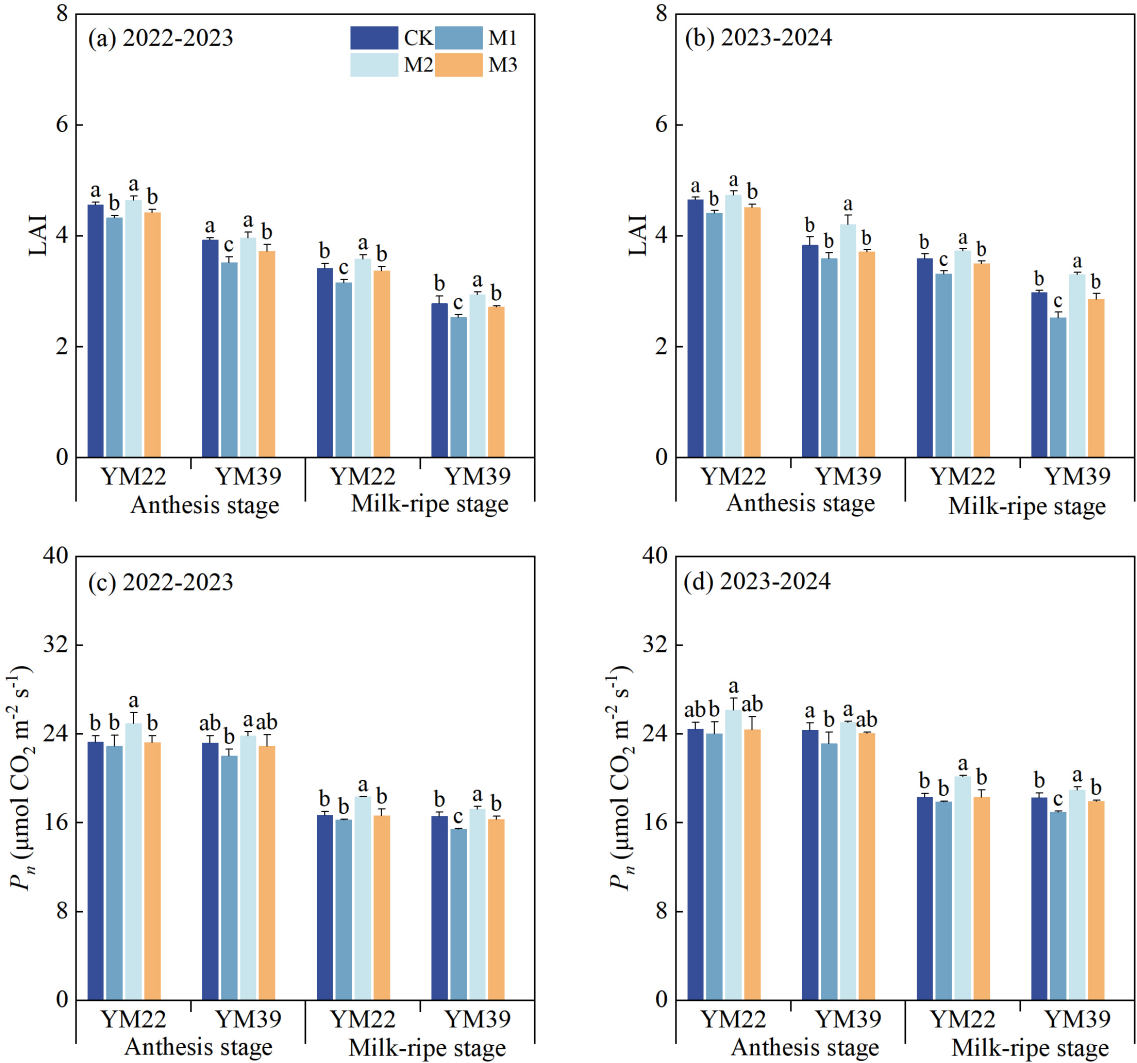
Effects of different deep application methods on leaf area index and photosynthetic rate of wheat at anthesis and milk-ripe stages. CK, split application of urea; M1, one-time broadcast application of SRNF combined with urea; M2, one-time deep band application of SRNF combined with urea; M3, one time alternate-row deep application of SRNF combined with urea. Vertical bars indicate mean standard errors of three replicates. Different lowercase letters indicate significant difference at 0.05 level.

As shown in **Figure 5**, compared with CK, the LAI of M1 was significantly reduced at the anthesis stage and milk-ripe stage. At the anthesis stage, the LAI decreases were 5.59% and 5.69% in the 2022–2023 and 2023-2024, respectively; at the milk-ripe stage, the LAI decreases were 8.19% and 11.41% in the 2022–2023 and 2023-2024, respectively. The LAI of M2 and M3 at the anthesis stage and milk-ripe stage was significantly higher than that of M1. At the anthesis stage, the LAI increases were 8.53%, 2.29% in 2022–2023 and 12.21%, 2.83% in 2023-2024, respectively; at the milk-ripe stage, the LAI increases were 13.11%, 4.70% in 2022–2023 and 21.64%, 4.84% in 2023-2024, respectively. There was no significant difference in the anthesis-stage LAI of M3 compared with that of M1 and CK. However, the milk-ripe stage LAI of M3 was significantly higher than that of M1, with increases of 4.70% and 4.84% in the 2022–2023 and 2023–2024 growing seasons, respectively. The milk-ripe stage LAI of M2 was significantly higher than that of CK, with increases of 3.84% and 7.43% in 2022–2023 and in 2023-2024, respectively. At the milk-ripe stage, there was no significant difference in LAI between M3 and CK.

As shown in **Figure 5**, compared with CK, Pn of M1 and M3 decreased slightly at the anthesis stage. The Pn of M2 and M3 at the anthesis stage was higher than that of M1, with M2 being significantly higher than M1. There was no significant difference in Pn between M2 and CK. Compared with M1, the Pn of M2 and M3 at the milk-ripe stage significantly increased, with increases of 20.52%, 11.68% and 21.01%, 12.08% in the 2022–2023 and 2023–2024 growing seasons, respectively. The Pn of M2 at the milk-ripe stage was significantly higher than that of CK, with increases of 6.99% and 7.56% in the 2022–2023 and 2023–2024 growing seasons, respectively. No significant difference in Pn at the milk-ripe stage was observed between M3 and CK.

### 3.5 Rhizosphere soil inorganic N content

**Figure 6:**
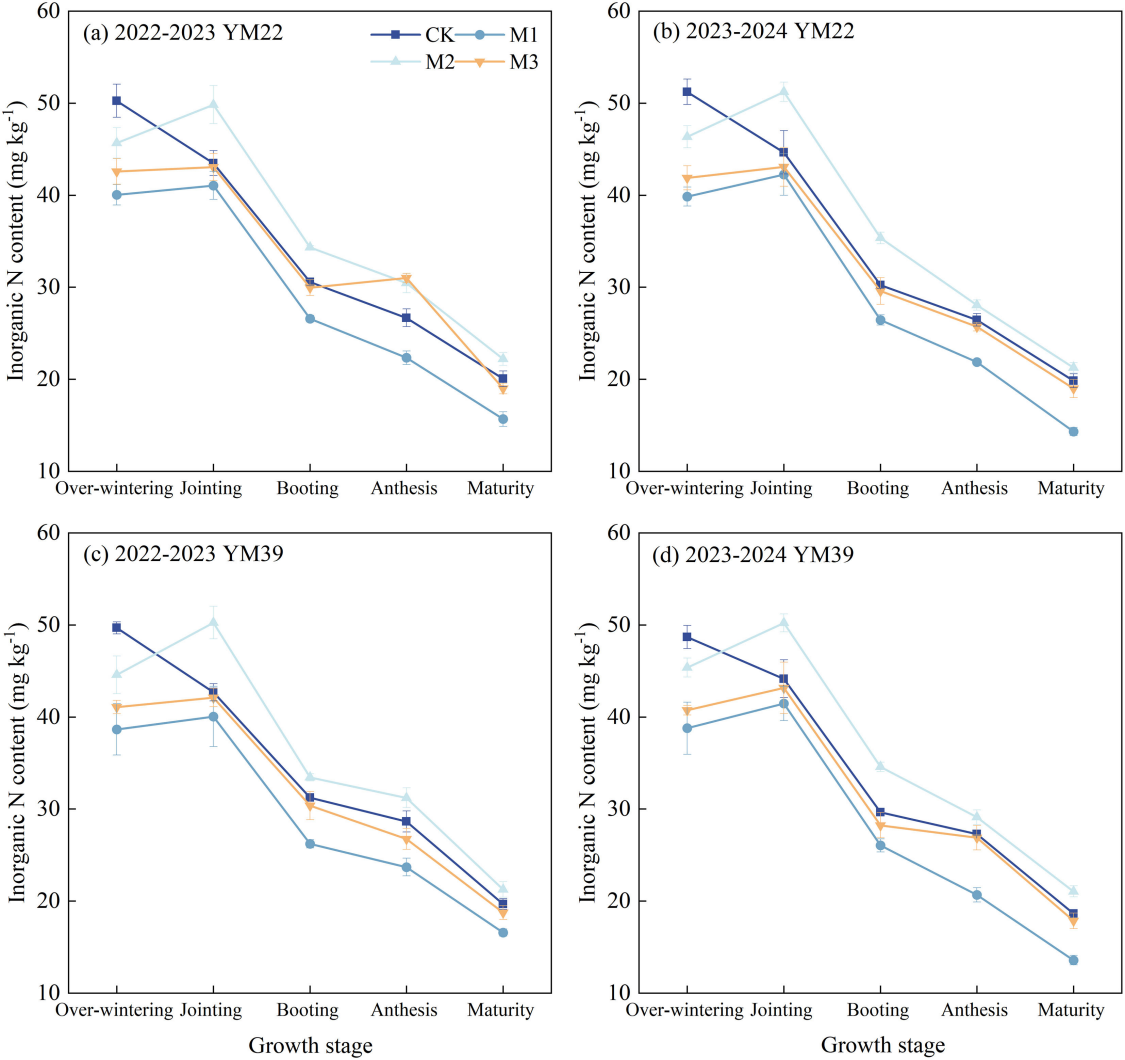
Effects of different deep application methods on wheat rhizosphere soil inorganic nitrogen content. CK, split application of urea; M1, one-time broadcast application of SRNF combined with urea; M2, one-time deep band application of SRNF combined with urea; M3, one time alternate-row deep application of SRNF combined with urea. Vertical bars indicate mean standard errors of three replicates. Different lowercase letters indicate significant difference at 0.05 level.

As shown in **Figure 6**, the rhizosphere soil inorganic N content of wheat under CK showed an overall decreasing trend throughout the entire growth period, while those under M1, M2, and M3 reached the maximum at the jointing stage and then gradually decreased. During the over-wintering stage, the overall trend of rhizosphere soil inorganic N content among all treatments was CK > M2 > M3 > M1, and the inorganic N content of CK was significantly higher than that of other treatments. At the jointing stage, the rhizosphere soil inorganic N content of M2 was significantly higher than that of other treatments, increasing significantly by 16.18% and 14.24% compared with CK in the 2022–2023 and 2023–2024 growing seasons, respectively. At this stage, the rhizosphere soil inorganic N content of CK was higher than that of M1 and M3, but there was no significant difference. During the booting stage, anthesis stage, and maturity stage, the rhizosphere soil inorganic N content of M2 was significantly higher than that of CK, with increases of 9.67% and 16.78% at the booting stage, 9.01% and 6.37% at the anthesis stage, and 9.40% and 9.96% at the maturity stage in the two growing seasons, respectively. In contrast, the rhizosphere soil inorganic N content of M3 was slightly lower than that of CK during the booting stage, anthesis stage, and maturity stage, but there was no significant difference between them.

### 3.6 N accumulation

**Figure 7:**
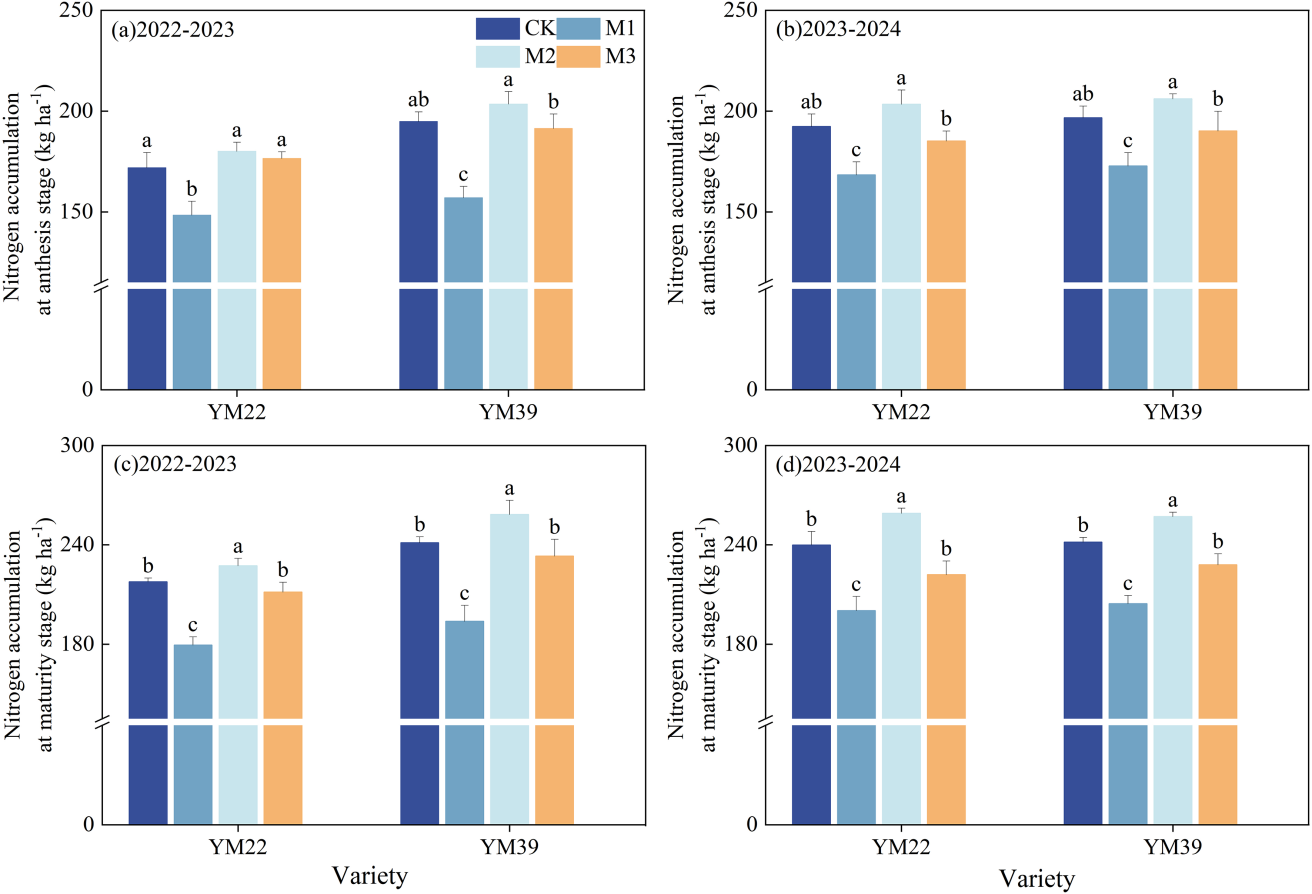
Effects of different deep application methods on N accumulation at anthesis and maturity. CK, split application of urea; M1, one-time broadcast application of SRNF combined with urea; M2, one-time deep band application of SRNF combined with urea; M3, one time alternate-row deep application of SRNF combined with urea. Vertical bars indicate mean standard errors of three replicates. Different lowercase letters indicate significant difference at 0.05 level.

As shown in **Figure 7**, compared with CK, M1 significantly reduced N accumulation at the anthesis stage and maturity stage. At the anthesis stage, the decreases in N accumulation were 16.53% and 12.32% in the 2022–2023 and 2023–2024 growing seasons, respectively; at the maturity stage, the decreases in N accumulation were 18.60% and 15.95% in the 2022–2023 and 2023–2024 growing seasons, respectively. Compared with M1, M2 and M3 significantly increased N accumulation at the anthesis stage and maturity stage. At the anthesis stage, the increases in N accumulation were 25.46%, 20.44% in 2022–2023 and 20.05%, 10.03% in 2023-2024, respectively; at the maturity stage, the increases in N accumulation were 30.00%, 19.08% in 2022–2023 and 27.53%, 11.18% in 2023-2024, respectively. There were no significant differences in N accumulation at the anthesis and maturity stages between M3 and CK.

**Figure 8:**
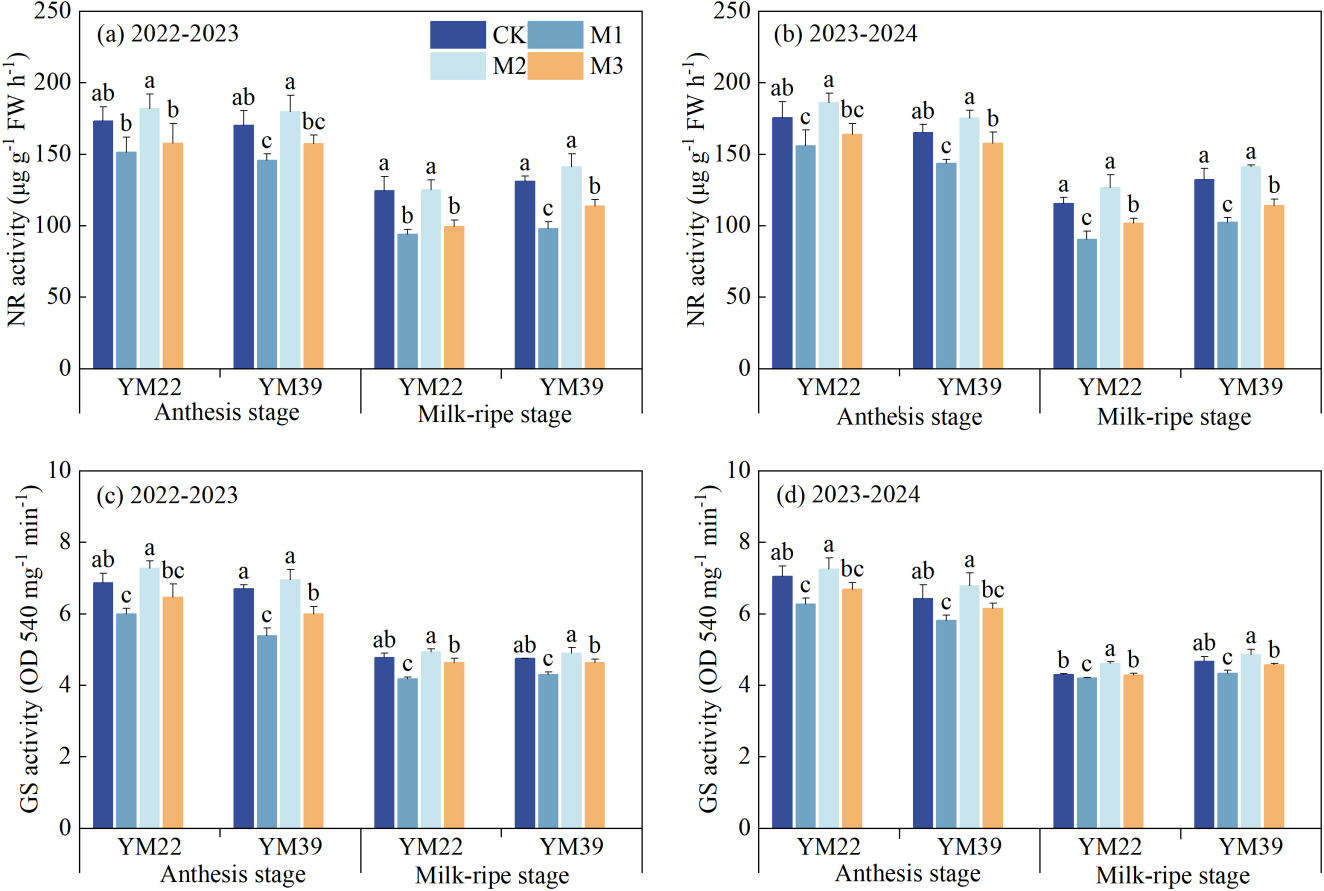
Effects of different deep application methods on NR and GS activities in flag leaves at anthesis and milk-ripe stages. CK, split application of urea; M1, one-time broadcast application of SRNF combined with urea; M2, one-time deep band application of SRNF combined with urea; M3, one time alternate-row deep application of SRNF combined with urea. Vertical bars indicate mean standard errors of three replicates. Different lowercase letters indicate significant difference at 0.05 level.

As shown in **Figure 8**, compared with CK, the activities of NR and GS in wheat under M1 and M3 treatments decreased at the anthesis stage, with M1 showing a significant difference from CK. Compared with M1, the NR and GS activities of M2 and M3 at the anthesis stage increased, among which M2 was significantly higher than M1. There was no significant difference in NR and GS activities between M2 and CK at the anthesis stage. Compared with CK, the NR and GS activities of M1 and M3 at the milk-ripe stage were significantly reduced: the NR activity decreased by 24.85% and 16.66% in 2022-2023, and by 22.08% and 12.75% in 2023-2024, respectively; the GS activity decreased by 11.12% and 2.83% in 2022-2023, and by 6.28% and 2.10% in 2023-2024, respectively. Compared with M1, the NR and GS activities of M2 and M3 at the milk-ripe stage were significantly increased: the NR activity increased by 38.61% and 10.92% in 2022-2023, and by 40.72% and 11.98% in 2023-2024, respectively; the GS activity increased by 16.09% and 9.35% in 2022-2023, and by 11.68% and 4.47% in 2023-2024, respectively. The NR and GS activities of M2 at the milk-ripe stage were significantly higher than those of CK: the NR activity increased by 4.15% in 2022–2023 and 9.66% in 2023-2024, respectively; the GS activity increased by 3.15% in 2022–2023 and 4.67% in 2023-2024, respectively. The NR and GS activities of M3 at the milk-ripe stage were lower than those of CK, but there was no significant difference.

As shown in **Table 4**, variety had an extremely significant effect on PNT, CPNT, and CPNA; treatment had an extremely significant effect on PNT, CPNT, PNA, and CPNA; and the interaction of variety and treatment (V×T) had an extremely significant effect only on CPNA. Compared with CK, M1 significantly reduced PNT, with decreases of 10.82% and 13.26% in the 2022–2023 and 2023–2024 growing seasons, respectively. Compared with M1, M2 and M3 increased PNT, with increases of 8.03%, 4.13% in 2022–2023 and 12.74%, 7.32% in 2023-2024, respectively. Compared with CK, the CPNT of M1 increased slightly, but the difference was not significant. Compared with M1, M2 significantly reduced CPNT, with decreases of 8.12% in 2022–2023 and 10.85% in 2023-2024, respectively. Compared with CK, M1 significantly reduced PNA and CPNA. For PNA, the decreases were 21.74% and 30.26% in the 2022–2023 and 2023–2024 growing seasons, respectively; for CPNA, the decreases were 9.70% and 15.12% in the 2022–2023 and 2023–2024 growing seasons, respectively. Compared with M1, M2 significantly increased PNA and CPNA. For PNA, the increases were 42.56%, in 2022–2023 and 73.12%, in 2023-2024, respectively; for CPNA, the increases were 22.58%, in 2022–2023 and 37.06%, in 2023-2024, respectively. The PNA of M3 was significantly lower than that of CK, with decreases of 16.84% and 22.12% in the 2022–2023 and 2023–2024 growing seasons, respectively.

**Table 4:** Effects of different deep application methods on N accumulation and remobilization in wheat.

Year	Variety	Treatment	Pre-anthesis N translocation (kg ha^-1^)	Contribution rate of pre-anthesis N translocation to the grain (%)	Post-anthesis N accumulation (kg ha^-1^)	Contribution rate of Post-anthesis N accumulation to the grain (%)
2022-2023	YM22	CK	107.04 ± 1.42a	69.01 ± 0.7ab	45.74 ± 0.22b	30.99 ± 0.46b
		M1	88.92 ± 4.94c	74.36 ± 4.12a	31.06 ± 0.44d	25.64 ± 0.97c
		M2	102.27 ± 2.01ab	67.25 ± 4.79b	47.28 ± 0.26a	32.75 ± 0.63a
		M3	100.38 ± 1.90b	73.20 ± 1.97ab	34.93 ± 0.35c	26.80 ± 1.19c
	YM39	CK	126.00 ± 5.11a	71.80 ± 1.77ab	46.38 ± 1.94b	28.20 ± 0.70b
		M1	107.57 ± 4.64c	72.56 ± 1.53a	36.75 ± 1.30d	27.44 ± 0.29c
		M2	120.22 ± 3.28ab	67.56 ± 1.53b	54.82 ± 0.90a	32.44 ± 0.33a
		M3	114.76 ± 4.05bc	72.51 ± 3.17a	41.72 ± 1.04c	27.49 ± 0.18c
2023-2024	YM22	CK	117.25 ± 1.24a	70.87 ± 2.68b	47.44 ± 2.45b	29.13 ± 0.90b
		M1	104.57 ± 1.23c	75.98 ± 1.92a	31.88 ± 1.16d	24.02 ± 0.91c
		M2	114.52 ± 2.71ab	68.72 ± 1.88b	55.60 ± 2.07a	31.28 ± 0.46a
		M3	113.00 ± 2.19b	75.84 ± 0.56a	36.74 ± 1.11c	24.16 ± 0.56c
	YM39	CK	135.18 ± 4.89a	75.99 ± 3.02ab	44.90 ± 1.26b	24.01 ± 0.69b
		M1	117.11 ± 2.75c	78.95 ± 0.87a	31.59 ± 1.63d	21.05 ± 1.03c
		M2	129.44 ± 3.26ab	71.43 ± 0.52b	50.80 ± 3.03a	28.57 ± 0.36a
		M3	123.07 ± 4.09bc	77.73 ± 1.48ab	37.74 ± 1.76c	22.27 ± 1.04c
LMM		V	**	*	ns	**
		T	**	**	**	**
		V×T	ns	ns	ns	**

CK, split application of urea; M1, one-time broadcast application of SRNF combined with urea; M2, one-time deep band application of SRNF combined with urea; M3, one time alternate-row deep application of SRNF combined with urea. In LMM results, * and ** indicate the significance of variety (V), treatment (T), and their interactions at the 0.05 and 0.01 levels, respectively; ns indicates no significant effect. Values are means ± standard error (n=3). Different lowercase letters after the same column value indicate significant difference at the level of 0.05.

### 3.7 NAE

**Figure 9:**
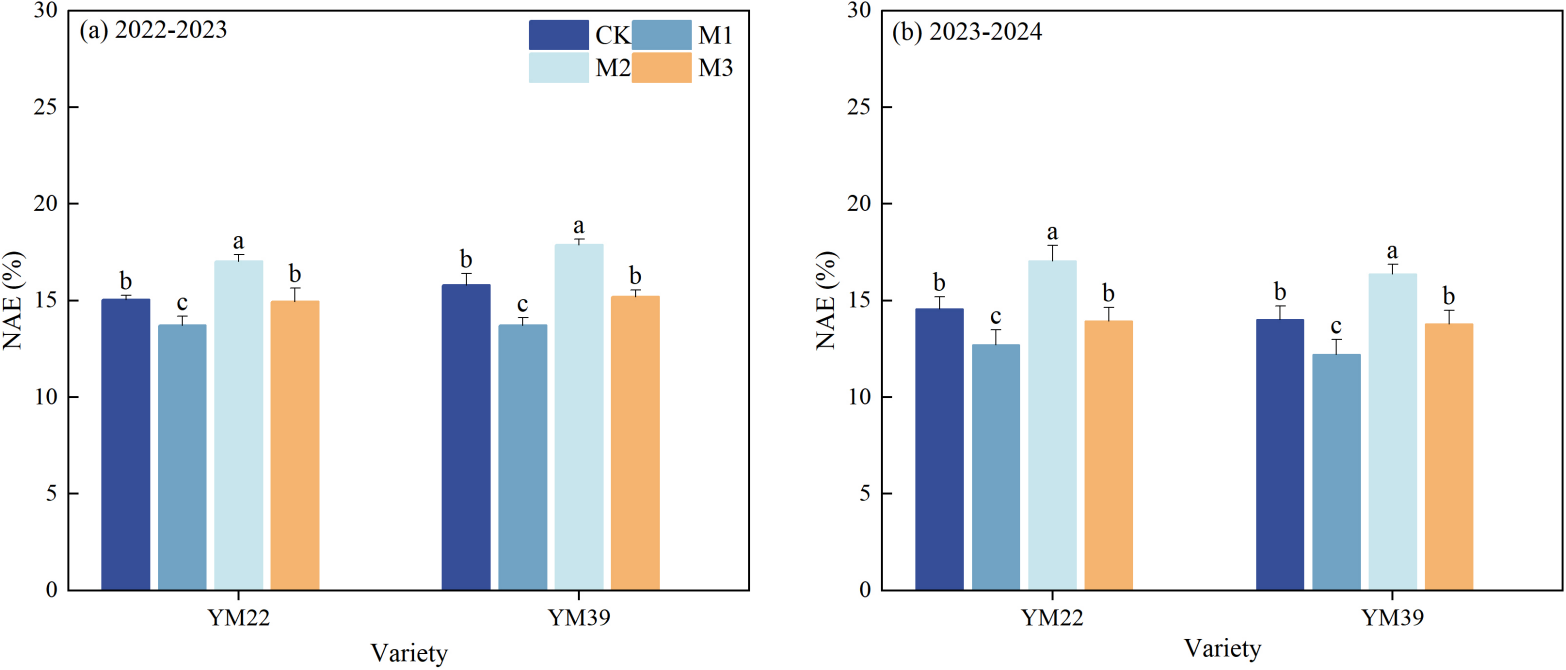
Effects of different deep application methods on N agronomic efficiency of wheat. CK, split application of urea; M1, one-time broadcast application of SRNF combined with urea; M2, one-time deep band application of SRNF combined with urea; M3, one time alternate-row deep application of SRNF combined with urea. Vertical bars indicate mean standard errors of three replicates. Different lowercase letters indicate significant difference at 0.05 level.

As shown in **Figure 9**, compared with CK, the NAE of M1 was significantly reduced, with decreases of 11.10% and 12.77% in the 2022–2023 and 2023–2024 growing seasons, respectively. The NAE of M2 and M3 was significantly higher than that of M1, with increases of 22.41%, 9.96% in 2022–2023 and 34.14%, 11.22% in 2023-2024, respectively. The NAE of M2 was significantly higher than that of CK, with increases of 13.20% in 2022–2023 and 17.00% in 2023-2024, respectively. There was no significant difference in NAE between M3 and CK.

### 3.8 Quality index

As shown in **Table 5**, variety had an extremely significant effect on PC, WGC, and SV; treatment had an extremely significant effect on PC and WGC, while it had a significant effect on SV. Compared with CK, the PC, WGC, and SV of M1 were all significantly reduced. For PC, the decreases were 13.37% and 13.38% in the 2022–2023 and 2023–2024 growing seasons, respectively; for WGC, the decreases were 8.62% and 17.37% in the 2022–2023 and 2023–2024 growing seasons, respectively; for SV, the decreases were 9.00% and 22.84% in the 2022–2023 and 2023–2024 growing seasons, respectively. The PC, WGC, and SV of M2 were all significantly higher than those of M1. For PC, the increases were 11.61% in 2022–2023 and 11.96% in 2023-2024, respectively; for WGC, the increases were 8.07% in 2022–2023 and 16.43% in 2023-2024, respectively; for SV, the increases were 6.87%, in 2022–2023 and 24.42% in 2023-2024, respectively. The PC, WGC, and SV of M2 were slightly lower than those of CK, but there was no significant difference. The PC, WGC, and SV of M3 were all significantly lower than those of CK. For PC, the decreases reached 10.30% and 10.19% in the 2022–2023 and 2023–2024 growing seasons, respectively; for WGC, the decreases reached 3.14% and 5.79% in the 2022–2023 and 2023–2024 growing seasons, respectively; for SV, the decreases reached 4.29% and 11.09% in the 2022–2023 and 2023–2024 growing seasons, respectively.

**Table 5:** Effects of different deep application methods on grain protein quality.

Year	Variety	Treatment	Protein content (%)	Wet gluten content (%)	Sedimentation value (mL)
2022-2023	YM22	CK	13.16 ± 0.53a	23.52 ± 0.29a	24.80 ± 0.65a
		M1	10.86 ± 0.24b	21.78 ± 0.23c	22.63 ± 0.34b
		M2	12.47 ± 0.47a	23.22 ± 0.30ab	24.06 ± 0.83a
		M3	11.65 ± 0.37b	22.86 ± 0.39b	23.67 ± 0.38ab
	YM39	CK	14.57 ± 0.56a	30.25 ± 0.09a	39.94 ± 0.15a
		M1	13.22 ± 0.50b	27.27 ± 0.61c	36.25 ± 0.69c
		M2	14.33 ± 0.57a	29.87 ± 0.33ab	38.94 ± 0.45ab
		M3	13.24 ± 0.54b	29.20 ± 0.51b	38.33 ± 0.90b
2023-2024	YM22	CK	13.00 ± 0.62a	25.55 ± 0.07a	29.41 ± 0.92a
		M1	11.22 ± 0.13b	21.17 ± 0.10c	23.92 ± 1.14c
		M2	12.46 ± 0.22a	25.04 ± 0.22ab	27.83 ± 0.67ab
		M3	11.73 ± 0.08b	24.36 ± 0.51b	25.84 ± 0.36bc
	YM39	CK	13.85 ± 0.22a	31.88 ± 0.67a	38.81 ± 1.02a
		M1	12.04 ± 0.38b	26.27 ± 0.84c	28.33 ± 1.10c
		M2	13.50 ± 0.34a	30.10 ± 1.21ab	37.59 ± 1.34ab
		M3	12.38 ± 0.60b	29.67 ± 1.11b	34.91 ± 2.29b
LMM		V	**	**	**
		T	**	**	*
		V×T	ns	ns	ns

M1, one-time broadcast application of SRNF combined with urea; M2, one-time deep band application of SRNF combined with urea; M3, one time alternate-row deep application of SRNF combined with urea. In LMM results, * and ** indicate the significance of variety (V), treatment (T), and their interactions at the 0.05 and 0.01 levels, respectively; ns indicates no significant effect. Values are means ± standard error (n=3). Different lowercase letters after the same column value indicate significant difference at the level of 0.05002E

### 3.9 Correlation analysis

**Figure 10:**
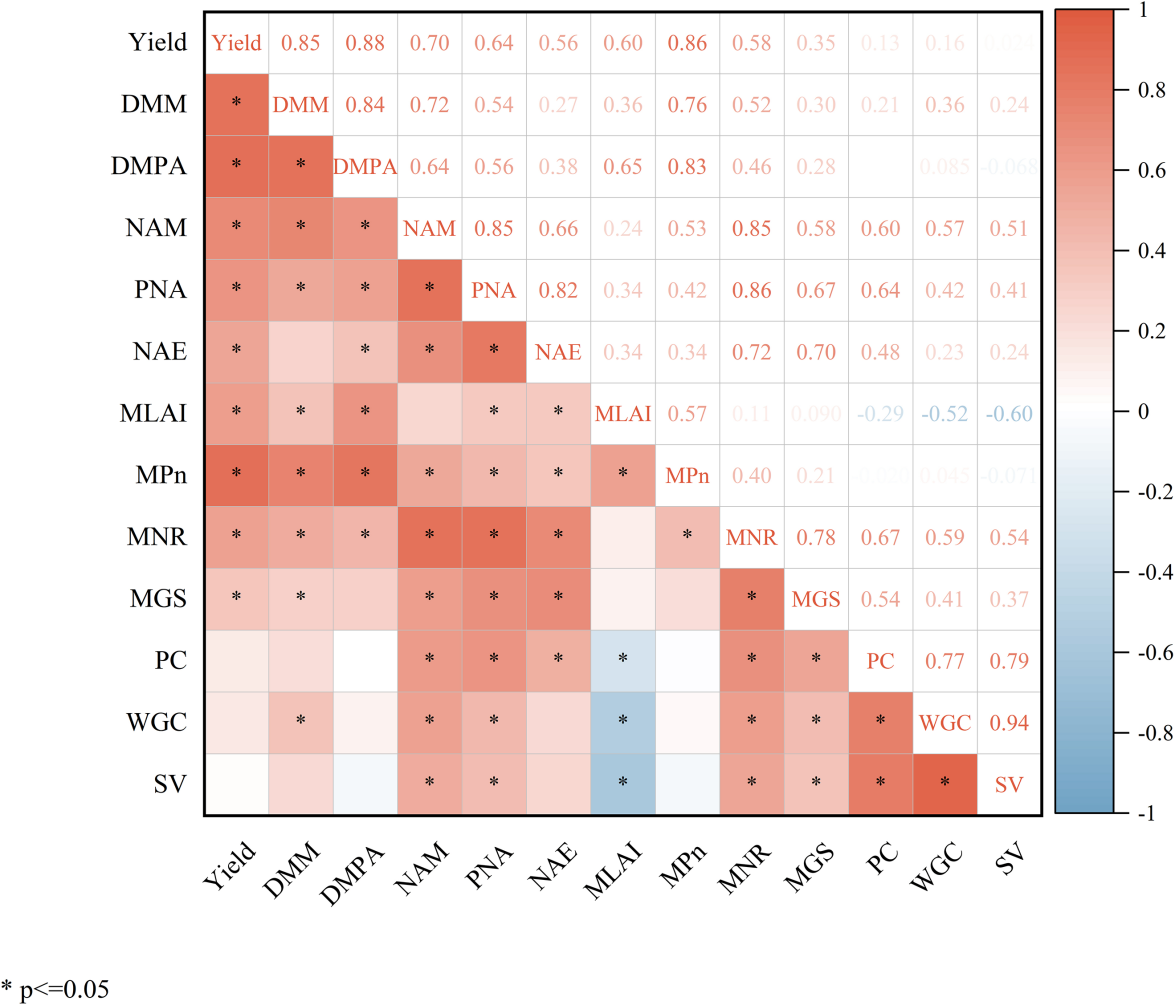
Correlation heatmap of wheat yield, nitrogen metabolism, net photosynthetic rate, and grain quality indexes. DMM, dry matter accumulation at maturity stage; DMPA, dry matter accumulation post-anthesis; NAM, nitrogen accumulation at maturity stage; PNA, post-anthesis nitrogen accumulation; NAE, nitrogen agronomic efficiency; MLAI, leaf area index at milk-ripe stage; M*P_n_*, net photosynthetic rate at milk-ripe stage; MNR, NR activity at milk-ripe stage; MGS, GS activity at milk-ripe stage; PC, protein content; WGC, wet gluten content; SV, sedimentation value. *Significant at the *p* < 0.05 level.

As shown in **Figure 10**, wheat yield showed a significant positive correlation with N accumulation at maturity, post-anthesis N accumulation, dry matter accumulation at maturity, post-anthesis dry matter accumulation, NAE, as well as LAI at the milk-ripe stage, and *P_n_*at the milk-ripe stage. Wheat yield also exhibited a significant positive correlation with NR activity at the milk-ripe stage, and GS activity at the milk-ripe stage. In addition, grain protein content, wet gluten index, and sedimentation value were all significantly positively correlated with N accumulation at maturity and post-anthesis N accumulation, while they were significantly negatively correlated with LAI at the milk-ripe stage.

## 4 Discussion

### 4.1 Effects of deep application of SRNF combined with urea on wheat yield and NAE

In wheat production, the type and application method of N fertilizer have a significant impact on yield (Duan et al., 2015; Shen et al., 2022). In this study, compared with the split application of urea treatment (CK), the one-time broadcast application of SRNF combined with urea treatment (M1) led to a significant decrease in grain yield (4.80%-5.99%) (**Table 1**). This result is consistent with the findings of Ma et al. (2021), that is, the one-time application of SRNF often has an unsatisfactory effect on wheat yield in the rice-wheat rotation system. Due to the broadcast application method in the M1 treatment, the risk of N fertilizer loss to the environment was increased, resulting in insufficient N supply in the later growth stage. This led to premature leaf senescence and a decrease in the net photosynthetic rate, reducing the dry matter accumulation, and ultimately resulting in a decrease in yield. In sharp contrast to the M1, the deep application of SRNF combined with urea (the M2 and M3 treatments) significantly increased wheat yield compared with CK (**Table 1**). However, there was no significant difference in grain yield between the M3 and CK treatments, but the M3 treatment effectively increased grain yield compared to the M1 treatment (**Table 1**). This result was consistent with previous studies, which found that deep application of urea or slow-release fertilizers increased the yields of crops such as maize and wheat compared with surface application (Wang et al., 2023; Wu et al., 2022). Wu et al. (2025) found that side-deep fertilization can place nutrients in the dense area of root growth during the seedling stage, enabling N to be quickly contacted and absorbed by seedling roots to promote early tillering. In this study, under deep application conditions, 30% urea in M2 and M3 was concentrated in the active root layer of wheat, which could improve the contact and absorption efficiency at the seedling stage compared with the M1 treatment, and promote early tillering (**Figure 3**). In addition, deep fertilization increased the N content in the rhizosphere soil of wheat during the middle and late growth stages (**Figure 6**), meeting the nutrient demand of the plants in these stages. This not only improved the tiller panicle rate but also maintained the grain filling intensity, which was conducive to the formation of the number of spikes and grains per spike. However, under the M3 treatment, one side of the wheat planting row was not fertilized, which might cause nutritional stress to some extent and be detrimental to the morphological development of the root system, and thus inhibited the absorption and utilization of fertilizer and reduced the yield of wheat plants compared to the M2 treatment.

A reasonable N supply is crucial for improving crop N accumulation and N use efficiency (Cassman et al., 2002; Raun and Johnson, 1999). In production, optimizing fertilization methods, rates, and timing can promote crop absorption of N, reduce nutrient loss, and thereby increase wheat yield (Ke et al., 2018; Li et al., 2012, 2021). In our study, the N accumulation of the M1 treatment at both anthesis and maturity stages was significantly lower than that of the CK treatment. Under the M2 and M3 treatments, the N accumulation level at maturity stage was obviously increased compared to the M1 treatment, with the M2 treatment significantly higher than the CK treatment (**Figure 7**). This might be mainly because deep application reduces N loss through surface runoff, ammonia volatilization, nitrous oxide emission, and other pathways (Akhtar et al., 2025; Duan et al., 2015; Ren et al., 2024.),. It not only lowers greenhouse gas emissions but also allows more N nutrients to be directionally absorbed and utilized by crops or retained in the soil (Zhang et al., 2022). In contrast, when fertilizers are applied shallowly, nutrients are distributed in the shallow plow layer, which easily leads to gaseous volatilization loss of N. On the other hand, the rhizosphere soil inorganic N content of wheat under Treatments M2 was significantly higher overall than that under the CK treatment after the jointing stage (**Figure 6**). As an important substrate for enzymatic reactions, the supply level of N directly affects enzyme activity (Yoneyama and Suzuki, 2019). Thus, sufficient N substrates in the M2 was beneficial for the maintaining high activities of N assimilation enzymes (NR and GS) compared to the M1 and CK treatments (**Figure 8**). This enabled the absorbed inorganic N to be effectively converted into organic N, ultimately improving N accumulation and N agronomic efficiency of wheat plants (**Figures 7**, **9**).

### 4.2 Effects of deep application of SRNF combined with urea on dry matter accumulation and remobilization of wheat

Dry matter accumulation is the foundation for wheat yield formation (Wang et al., 2025). The Contribution rate of dry matter remobilized pre-anthesis to the grain accounts for approximately one-third of grain yield, while the Contribution rate of dry matter accumulated post-anthesis to the grain exceeds two-thirds (Yan et al., 2022; Zhu et al., 2024). Compared with CK, the PDRA in the M1 treatment was significantly increased. This may be attributed to the low *P_n_* in the late growth stage, which led to insufficient supply of assimilates during the grain filling period and thus accelerated the remobilization of stored substances in pre-anthesis vegetative organs. However, the CDP to the grain is limited and cannot fully compensate for the deficiency in post-anthesis assimilate supply. Therefore, the yield under the M1 treatment was still significantly lower than that under the CK treatment (**Table 1**). In contrast, although the M2 treatment reduced the PDRA compared with CK, it significantly increased the PDMA of wheat (**Table 3**). For the M3 treatment, there was no significant difference in PDMA compared with CK, but it was significantly higher than that under the M1 treatment. This may be due to the deep application of SRNF combined with urea, which maintained high activities of N metabolism-related enzymes in wheat at both the anthesis and milk-ripe stages, thereby promoting N uptake and assimilation. This not only helps delay leaf senescence but also maintains the integrity of photosynthetic structures, ultimately facilitating PDMA and laying a solid material foundation for the improvement of wheat yield (Wang et al., 2023b). Further correlation analysis showed that dry matter accumulation, N accumulation, photosynthetic characteristics, and N metabolism-related enzyme activities were all significantly positively correlated with wheat yield and NAE (**Figure 10**). This result indicates that the deep application of SRNF combined with urea improved the N accumulation level of wheat in the middle and late growth stages, thereby enhancing carbon assimilation efficiency and promoting the synergistic improvement of carbon and N metabolism, ultimately facilitating grain yield formation.

### 4.3 Effects of deep application of SRNF combined with urea on wheat protein quality

The protein content in wheat grains is jointly regulated by pre-anthesis N translocation and post-anthesis N uptake (Kichey et al., 2007). Previous studies have shown that the contribution rate of pre-anthesis N translocation to the total N in grains is 70%, while that of PNA is only 30% (Zhang et al., 2022). Although the contribution ratio of PNA to the total N in grains is not high, its contribution rate to grain protein quality is crucial (Li et al., 2021a). Correlation analysis showed that grain protein content was significantly positively correlated with both N accumulation at maturity and post-anthesis N accumulation (**Figure 10**). Compared with the CK treatment, the PC, WGC and SV in the M1 treatment were significantly decreased (**Table 5**), mainly due to its lower N accumulation pre- and post-anthesis (**Table 4**). The M2 treatment increased PNA and CPNA compared to the M1 and CK treatments by stabilizing N supply during the late growth stages, thereby increasing wheat grain protein content (**Tables 4**, **5**). This was beneficial to maintaining the quality stability of YM39, a medium-strong gluten wheat variety. However, for weak gluten wheat, the higher protein content and wet gluten content in the M2 treatment were unfavorable to the formation of weak gluten wheat quality. Under the condition that the M3 yield did not decrease significantly compared with CK, this treatment reduced PNA in wheat aboveground parts, resulting in a significant decrease in grain PC, WGC and SV (**Table 5**), which had an obvious improvement effect on the quality of YM22, a weak gluten wheat variety. The M3 treatment involved one-sided fertilization for wheat, which induced roots to grow toward the fertilization side. In this case, there was no intense spatial competition between roots due to low root biomass in the early growth stages. However, with the continuous increase of root biomass, the spatial competition between roots would intensify, especially in the late growth stage, and this intensification caused premature senescence of roots and decreased N uptake and accumulation. This might be the main reason why the M3 treatment significantly reduced PNA compared to the M2 and CK treatments.

## 5 Conclusion

Our study found that the one-time deep application technology of SRNF combined with urea can improve the N environment in the rhizosphere soil, thereby promoting N uptake and photosynthetic capacity in the above-ground parts, and ultimately achieving increased wheat yield. Therefore, this technology can serve as an alternative to the conventional split application of N fertilizer in the middle and lower reaches of the Yangtze River. For wheat varieties with different gluten types, growers can select a one-time deep application method of SRNF combined with urea that is suitable for their specific gluten type. However, future research needs to explore the impact of this technology on the environment and the specific morphological responses of roots, as well as verify the generalizability of the results; in addition, efforts should be made to strengthen the research and development of supporting fertilization machinery and equipment to meet the needs of technology promotion.

## Data Availability

The original contributions presented in the study are included in the article/supplementary material. Further inquiries can be directed to the corresponding author/s.
